# Airway and anesthesia management in tracheoesophageal fistula closure implantation: a single-centre retrospective study

**DOI:** 10.1186/s13019-024-02737-4

**Published:** 2024-04-03

**Authors:** Zhu Dechong, Huang He, Zhang Jigang, Liu Cunming

**Affiliations:** Department of Anesthesiology and Perioperative Medicine, the First Affiliated Hospital of Nanjing Medical University, Nanjing, 210029 China

**Keywords:** Tracheoesophageal fistula, Occlusion, Gastroscopy, Fiberoptic bronchoscope, Anesthesia management

## Abstract

**Objective:**

To review and analyze the airway and anesthesia management methods for patients who underwent endoscopic closure of tracheoesophageal fistula (TEF) and to summarize the experience of intraoperative airway management.

**Method:**

We searched the anesthesia information system of the First Affiliated Hospital of Nanjing Medical University for anesthesia cases of TEF from July 2020 to July 2023 and obtained a total of 34 anesthesia records for endoscopic TEF occlusion. The intraoperative airway management methods and vital signs were recorded, and the patients’ disease course and follow-up records were analyzed and summarized.

**Results:**

The airway management strategies used for TEF occlusion patients included nasal catheter oxygen (NCO, *n* = 5), high-flow nasal cannula oxygen therapy (HFNC, *n* = 4) and tracheal intubation (TI, *n* = 25). The patients who underwent tracheal intubation with an inner diameter of 5.5 mm had stable hemodynamics and oxygenation status during surgery, while intravenous anesthesia without intubation could not effectively inhibit the stress response caused by occluder implantation, which could easily cause hemodynamic fluctuations, hypoxemia, and carbon dioxide accumulation. Compared with those in the TI group, the NCO group and the HFNC group had significantly longer surgical times, and the satisfaction score of the endoscopists was significantly lower. In addition, two patients in the NCO group experienced postoperative hypoxemia.

**Conclusion:**

During the anesthesia process for TEF occlusions, a tracheal catheter with an inner diameter of 5.5 mm can provide a safe and effective airway management method.

## Introduction

Tracheoesophageal fistula (TEF) is a pathological connection between the esophagus and the trachea or bronchus that can occur after surgery, radiation, chemotherapy or airway invasion. Previous studies have indicated that TEF occurs in approximately 5–15% of patients due to esophageal malignancies and 1% due to bronchial cancer. In addition, prolonged intubation is a growing cause of benign acquired tracheoesophageal fistula [1–3]. Due to the lack of effective treatment for TEF fistulas, patients often experience respiratory failure combined with other serious complications, which significantly reduces their quality of life and increases their disability fatality rate [4]. At present, there is no standard treatment for TEF; although there have been cases of thoracoscopic repair of TEF involving muscle, pericardial or thymic valves [5], most TEF patients cannot tolerate surgery due to malnutrition combined with pulmonary complications.

In recent years, endoscopic TEF repair methods, such as self-expanding metal stents, degradable stents, silicone stents, and unidirectional umbrella valves in the bronchi, have been increasingly widely applied for the repair of TEF fistulas [6, 7]. Since endoscopic TEF repair is more characterized by individualized treatment, there is no standardized program for anesthesia management. Based on the design concept of congenital heart disease occluders, the gastroenterology team of our hospital independently developed new double-disc digestive tract fistula occluders (hereinafter referred to as occluders) in 2020; these occluders can be safely and effectively used for TEF repair and significantly improve patients’ quality of life [8]. We used nasal catheter oxygen and high-flow oxygen sedation anesthesia to complete 9 occlusive device implantation procedures, but these anesthesia methods were abandoned because they cannot effectively prevent stress reactions and maintain stable vital signs. Finally, we adopted general anesthesia with 5.5 mm endotracheal intubation. In this retrospective study, we analyzed the case data of patients who underwent TEF occluder implantation, aiming to summarize the characteristics of airway management during anesthesia and share the experience of success or failure.

## Data and methods

This study was approved by the Ethics Committee of the First Affiliated Hospital of Nanjing Medical University (approval number: 2023-SR-088). Anesthesia-treated patients with TEF from July 2020 to July 2023 were identified in the information system of the First Affiliated Hospital of Nanjing Medical University, and a total of 34 records of TEF occlusion were obtained.

### Operative procedures

Occluder implantation at our center requires combined gastroscopy and tracheoscopy. The implant is placed into the fistula through endoscopic forceps under direct vision of the gastroscope. Under direct vision of the fiberoptic bronchoscope, biopsy forceps are used to assist in the release of the occluder on the tracheal side. After confirming the completion of bilateral occlusion, 0.2% methylene blue is sprayed on the fistula opening on the gastroscope side to observe whether there is leakage and to determine whether the occluder is successfully placed [7].

### Methods of anesthesia

**Fig. 1:**
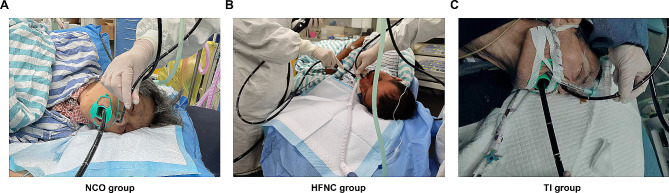
Intraoperative oxygen supply in three groups

According to the description of the anesthesia records, all patients were anesthetized via the respiratory surface by aerosol inhalation of local anesthetics. Patients in the NCO group received 5–8 L/min oxygen inhalation through a nasal catheter (Fig. 1A). Patients in the high-flow nasal cannula oxygen therapy (HFNC) group were given 45–60 L/min oxygen inhalation (Fig. 1B). After full oxygen inhalation and nitrogen removal, 1-1.5 mg/kg propofol combined with 0.2–0.5 µg/kg remifentanil was injected intravenously. The operation started when the patients’ MOAA/S sedation score reached 2–3. During the operation, propofol was continuously administered intravenously at 50–70 µg/(kg·min), and RF was pumped intravenously at 0.3–0.6 µg/(kg·min) to maintain the appropriate depth of anesthesia.

To avoid reflux aspiration caused by positive mask pressure ventilation during induction, gastroscopy was performed before anesthesia in the tracheal intubation group. After full oxygen inhalation and nitrogen removal, 0.05 mg/kg midazolam, 0.2 mg/kg etomidate, 0.2 µg/kg sufentanil and 0.6 mg/kg rocuronium were successively injected intravenously. In addition, 2% lidocaine was sprayed on the glottis via a fiberoptic bronchoscope. After the diameter of the tracheal catheter was recorded, it was found that all the patients had tracheal catheters with an internal diameter of 5.5 mm (Fig. 1C). With the help of a fiberoptic bronchoscope, it was determined that the end of the tracheal catheter was located above the carina, and the cuff was passed through the fistula. During the operation, mechanical ventilation was performed in volumetric control mode, and the inhaled O_2_ concentration (FiO_2_) was set at 60%. The intraoperative intravenous pump dose of propofol was 60–80 µg/(kg·min), and the remifentanil pump dose was 0.5–0.8 µg/(kg·min).

### Observation indicators

The corresponding case information was obtained from the hospital case information system, the general information of patients, the anesthesia protocol (airway management), intraoperative hemodynamics, the operation satisfaction score, follow-up results, etc. The obtained data were analyzed and summarized.

### Statistical analysis

GraphPad 8.0 software was used for statistical analysis. The measurement data did not conform to a normal distribution and are expressed as M (0.25, 0.75); the Kruskal‒Wallis test was used. The measurement data are expressed as the mean ± standard deviation (SD); count data are expressed as an example. *P* < 0.05 was considered to indicate statistical significance.

## Results

The data of 34 patients were summarized according to different anesthesia management schemes for TEF occlusions under endoscopy. A total of 9 patients received intravenous anesthesia, including 5 patients in the NCO group and 4 patients in the HFNC group. A total of 25 patients underwent endotracheal intubation under general anesthesia. The general clinical data of the three groups of patients are shown in Table 1.

The diameter of the fistula and the causes of TEF formation are presented in Table 1. According to the recorded data from the three groups, the distance between the fistula and the carina was 3–5 cm.

**Table 1:** General clinical data of patients in the three groups

Observational index	NCO group (*n* = 5)	HFNC group (*n* = 4)	TI group (*n* = 25)
Male/female	4/1	3/1	18/7
Age (years)	59.8 ± 7.3	57.3 ± 18.7	61.0 ± 9.3
BMI(kg/m^2^)	18.3 ± 0.8	18.2 ± 0.7	17.9 ± 0.6
ASA II/III	1/4	1/3	10/15
Pulmonary infection	3	3	16
Diameter of TEF (cm)	1.3 ± 0.2	1.4 ± 0.4	1.3 ± 0.1
The distance between fistula and carina (cm)	3.96 ± 0.34	3.7 ± 0.42	3.78 ± 0.39
Causes of TEF formation			
Post operation of esophageal	4	3	20
Tracheotomy or trauma	1	1	5

NCO: nasal catheter oxygen; HFNC: high-flow nasal cannula oxygen therapy; TI: tracheal intubation; TEF: tracheoesophageal fistula; BMI: body mass index; ASA: American *Society of Anesthesiologists*

In this study, we analyzed the changes in vital signs in each group at different time points. There were no significant differences in mean arterial pressure (MAP), heart rate (HR) or pulse oxygen saturation (SpO_2_) among the groups before induction of anesthesia or before surgery (*P* > 0.05). At the release time point of the occluder, the MAP and HR were significantly greater in the NCO group and HFNC group than in the TI group (*P* < 0.05); moreover, the SpO_2_ in the NCO group was significantly lower than that in the TI group and the HFNC group (*P* < 0.05). Notably, 2 patients had suspended the operation due to hypoxemia during surgery and needed to improve oxygenation status through assisted ventilation.

Compared with those before occluder implantation, the MAP and HR were significantly greater in the NCO group and the HFNC group when the occluder was released (*P* < 0.05), while the SpO_2_ was significantly lower in the NCO group (*P* < 0.05), as shown in Table 2. These results suggest that intravenous anesthesia cannot effectively control the stress response during occluder implantation, which easily causes intraoperative hemodynamic fluctuations and significantly increases the incidence of hypoxia, while tracheal intubation under general anesthesia can effectively control surgical stress and maintain the stability of intraoperative hemodynamics and oxygenation.

Arterial blood gas levels were analyzed immediately after occluder implantation, and the results showed that the PaO_2_ concentration was 125.3 ± 7.7 mmHg and the PaCO_2_ concentration was 56.8 ± 3.1 mmHg in the NCO group. The PaO_2_ and PaCO_2_ were 178.5 ± 6.9 mmHg and 53.9 ± 3.4 mmHg, respectively, in the HFNC group. In the TI group, the PaO_2_ was 226.8 ± 8.1 mmHg, and the PaCO_2_ was controlled at 41.5 ± 2.7 mmHg. These results suggest that the use of nasal catheters or nasal high-flow oxygen inhalation during occluder implantation increases the risk of carbon dioxide accumulation, while intubation under general anesthesia can effectively maintain the level of arterial blood partial pressure of carbon dioxide.

**Table 2:** Comparison of vital signs among the three groups at different time points

	Before induction of anesthesia	Post-anesthesia induction	Occluder release time	End of operation
*MAP*				
NCO group	91 (82, 100)	78 (74, 80)	84 (80, 87.5)	82 (75, 84.5)
HFNC group	92 (83.5, 100.5)	79 (75.75, 81.5)	84 (82.5, 86.25)	81.5 (71.25, 88.75)
TI group	91 (82, 104.5)	81 (72.5, 84)	75 (70, 79)	81 (77, 82)
*P* value	0.92	0.59	0.0034	0.71
*HR*				
NCO group	86 (82.5, 89.5)	76 (70, 78.5)	87 (81.5, 99.5)	89 (79.5, 90)
HFNC group	90 (75.5, 94.75)	73 (68.75, 92.25)	92 (83.75, 95)	83 (75.75, 90.25)
TI group	90 (80.5, 99.5)	78 (71, 80)	79 (70.5, 84.5)	81 (73, 92)
*P* value	0.83	0.94	0.0132	0.74
*SpO*_*2*_				
NCO group	97 (96, 98)	99 (98, 99.5)	96 (90.5, 98)	97 (96, 99)
HFNC group	97 (95.25, 98)	99 (98.25, 99.75)	98 (96.5, 99.5)	98.5 (95, 99.75)
TI group	97 (97, 99)	100 (99, 100)	99 (98, 100)	99 (99, 99)
*P* value	0.11	0.11	0.0075	0.12

MAP: mean arterial pressure; HR: heart rate; SpO_2_: pulse oxygen saturation; NCO: nasal catheter oxygen; HFNC: high-flow nasal cannula oxygen therapy; TI: tracheal intubation;

Compared with that in the TI group, the operation time of patients in the NCO group and the HFNC group was significantly longer (*P* < 0.05), and the satisfaction score of the endoscopic physician was significantly lower (*P* < 0.05). Hypoxemia (SpO_2_ < 92%) occurred in 2 patients in the NCO group. There was no significant difference in the number of days of hospitalization between the two groups (*P* > 0.05) (Table 3).

**Table 3:** Comparison of perioperative data among the three groups

Group	Time of operation (min)	Operation satisfaction score	Hypoxemia	Postoperative hospital stay
NCO group	58 (52, 62)	3 (2, 3)	2	2.5 (2, 3)
HFNC group	56.5 (54.25, 59.5)	3 (2.25, 3)	0	3 (2, 3)
TI group	46 (43, 50)	5 (4, 5)	0	3 (2.5, 3.5)
*P* value	0.0002	< 0.0001		0.47

NCO: nasal catheter oxygen; HFNC: high-flow nasal cannula oxygen therapy; TI: tracheal intubation

## Discussion

At present, the treatment methods for TEF mainly include surgical treatment, interventional therapy and conservative medical treatment [9]. Patients with intractable TEF cannot tolerate thoracoscopic or thoracoscopic surgery due to severe lung infection, malnutrition or other conditions [10]. With the development of endoscopy technology, new progress has been made in the minimally invasive treatment of TEF. However, there is no unified anesthesia protocol for TEF repair under endoscopy, and intraoperative airway management has also created great challenges for anesthesiologists. Therefore, the exploration of safe and effective airway management protocols will contribute to the development of endoscopic TEF therapy.

In recent years, a new double-disc digestive tract fistula plugging device designed by Zhang Guoxin et al. has been used to treat TEF. This device releases the double mushroom head stent under direct vision from the gastroscope and fiberbronchoscope to occlude the fistula on the esophageal side and tracheal side [11]. Given that the patients were complicated with frailty and pulmonary complications, we explored the anesthesia method for these patients, and intravenous anesthesia without intubation was initially considered. However, when occluder implantation was combined with gastroscopy and bronchoscopy, the stimulation of the esophagus and airway increased, and the results of this study showed that the blood pressure and heart rate of patients in the NCO group significantly increased at the time of release of the occluder. When the depth of anesthesia was increased to inhibit the cardiovascular stress response, the operation was suspended in 2 patients in the NCO group due to hypoxemia, and the oxygenation status was improved by assisted ventilation.

A previous study showed that HFNC therapy could significantly reduce the risk of hypoxemia in patients receiving gastroenteroscopy under sedation anesthesia [12]; therefore, we applied HFNC therapy in endoscopic TEF therapy. The results showed that HFNC intravenous anesthesia could not effectively inhibit the cardiovascular stress response during the release of the occluder. In addition, we found that when the operation time of endoscopic TEF occlusion was more than half an hour, HFNC therapy could also not maintain oxygenation during the operation. These results suggest that nonintubated intravenous anesthesia does not effectively inhibit the stimulation of occluder implantation under combined gastroscopy and bronchofiberscopy; moreover, it increases the risk of hypoxemia and carbon dioxide accumulation. In addition, nonintubation sedative anesthesia cannot prevent airway obstruction caused by airway bleeding during endoscopic operation. Therefore, only 9 patients in our center underwent catheterless intravenous anesthesia to complete occluder implantation.

Previous studies have reported that tracheoesophageal fistulas are blocked by tracheal stents through laryngeal masks under general anesthesia [13]. In this case, the stent was only released under a fiberbronchoscope, and a gastroscope was not used simultaneously. The use of a laryngeal mask will affect the effectiveness of combined gastroscopy and bronchoscopy when the new TEF occluder is used; therefore, we did not consider the use of a laryngeal mask. Tracheal intubation under general anesthesia is more commonly used in the surgical treatment of TEF. Due to the characteristics of the double mushroom head structure of the device, a fiberbronchoscope requires the use of biopsy forceps to assist in the release of the occlusal device under the fiberbronchoscope when releasing the occlusal device. Since the fiberbronchoscope requires sufficient maneuvering space outside the tracheal catheter and because the fiberbronchoscope and tracheal catheter must share the same airway, the selection of tracheal catheter type during general anesthesia should be carefully evaluated.

If a tracheal catheter with an internal diameter of 7.0–7.5 mm is selected, a fiber bronchoscope cannot be effectively combined with a gastroscope to complete the implantation of the occluder when the airway is entered through the tracheal catheter, while a fiber bronchoscope is convenient for observing the fistula and facilitating the operation. Tracheal anatomy revealed that the diameter of the male trachea was 1.2–1.5 cm, whereas that of the female trachea was 1.0–1.2 cm. Considering that the diameter of the fiberbronchoscope is approximately 0.5–0.6 cm, the use of a tracheal catheter with an inner diameter of 5.5 mm can not only ensure ventilation but also provide enough space for the fiberbronchoscope to operate outside the catheter to guide accurate implantation of the occlusive device more effectively.

The results of this study showed that general anesthesia could not only effectively inhibit the stress response caused by combined gastrectomy and fiberbronchoscopy, maintaining stable hemodynamics during the operation but also ensuring the stable oxygenation status of patients and avoiding carbon dioxide accumulation. All patients were successfully extubated, and no postoperative hypoxemia occurred. In addition, compared with surgery under nonintubated intravenous anesthesia, endotracheal catheterization under general anesthesia can prevent operation interruption caused by unstable vital signs during surgery, significantly improve patient satisfaction with the endoscope, and shorten patient operation time, which is a significant benefit for patients with intractable TEF. This retrospective study has several limitations. First, the number of patients included was limited, and a large sample and prospective study are needed to verify the effectiveness and safety of this airway management protocol. Second, the fistulas of the patients in this study were located on the protuberance, so the safety of this protocol for subprotuberance TEF treatment needs to be evaluated.

In summary, airway management in patients with endoscopic TEF tumors remains challenging. Gastroscopy combined with fiberbronchoscopy for occluder implantation brings new hope for the treatment of refractory TEF. This study provides a clinical basis for the safe application of a 5.5 mm long endotracheal catheter in the implantation of new TEF occluders.

## Data Availability

The datasets generated and/or analyzed during the current study are not publicly available due to policy issues in the hospital but are available from the corresponding author on reasonable request.
